# Clinicopathological characteristics and loss of mismatch repair protein expression in Chinese upper tract urothelial carcinomas

**DOI:** 10.3389/fonc.2022.1012168

**Published:** 2022-10-25

**Authors:** Zhi Shang, Shengming Jin, Wenwen Wang, Yu Wei, Chengyuan Gu, Chen Yang, Yu Zhu, Yao Zhu, Yijun Shen, Junlong Wu, Dingwei Ye

**Affiliations:** ^1^ Department of Urology, Fudan University Shanghai Cancer Center, Shanghai, China; ^2^ Department of Oncology, Shanghai Medical College, Fudan University, Shanghai, China; ^3^ Department of Urology, Huashan Hospital, Fudan University, Shanghai, China

**Keywords:** upper tract urothelial carcinoma, mismatch repair proteins, immunohistochemistry, East Asia, urothelial carcinoma

## Abstract

Expression of DNA mismatch repair (MMR) protein (MLH1, PMS2, MSH2, and MSH6) in upper tract urothelial carcinoma (UTUC) has been explored in Western cohorts, but it is rarely reported in Eastern cohorts. We aimed to assess the loss of MMR protein expression among Chinese UTUC patients and study its clinicopathological implications. We enrolled 175 UTUC patients at our center and tested the expression of MMR proteins by immunohistochemistry. Then, we explored these patients’ clinicopathological characteristics. We found loss of MMR proteins in 19 (10.9%) of 175 patients in our cohort (6 MSH2 and MSH6, 2 MSH6 alone, 6 MSH2 alone, 3 MLH1 and PMS2, and 2 PMS2 alone). Loss of MMR proteins was not a significant prognostic factor of relapse-free survival for these patients. In addition, patients with lower T stage or with bladder cancer history were more likely to have loss of MMR protein expression. At last, two metastatic patients (MSH2 and MSH6 loss; MSH2 loss) with loss of MMR protein experienced tumor recession after several cycles of anti-PD-1 immunotherapy. In conclusion, this is the largest Chinese UTUC cohort study to date that explores the loss of MMR protein expression. The rate of MMR loss observed was comparable to that in the Western UTUC cohort, supporting universal UTUC screening in China. Furthermore, a subset of advanced UTUCs with MMR protein loss are probably immunogenic, for whom single or combined immunotherapy may be potential therapeutic options in the future.

## Introduction

Urothelial carcinoma (UC) is the sixth most common cancer in America (1). Urothelial carcinoma of the bladder (UCB) takes up 90%–95% of UC (2). Compared with UCB, upper tract urothelial carcinoma (UTUC) is a rare malignant tumor, accounting for 5%–10% of UCs (1, 3). UTUC normally originates in the renal pelvis and ureter.

Lynch syndrome (LS) is defined by an autosomal dominant tumor syndrome that is regarded as the loss of expression of one of the DNA mismatch repair (MMR) genes including MLH1, MSH2, MSH6, and PMS2 (4, 5). Alterations in MMR genes often result in mutations that can be more vulnerable to a diversity of malignancies, especially colorectal carcinomas, endometrial carcinomas, and UTUCs (6).

However, universal cancer screening for LS is only suggested for patients with colorectal or endometrial carcinoma by detecting loss of MMR expression (7–9). Although UTUC is the third most common malignancy in patients with LS, it is often neglected (10) Approximately 2% (11) to 11% (12) of patients with UTUC were reported to have loss of expression of MMR. Thus, in clinical practice, doctors should pay more attention to UTUC in patients with LS (13). In addition, it is of great importance to perform universal screening for LS by detecting expression of MMR proteins in UTUC patients.

Approximately 70.7% (14) to 100% (15) UTUC patients were observed to develop a high-grade tumor eventually, with a worse prognosis (16). The 5-year survival rate of patients with pT2–pT3 is fewer than 60% and that of patients with pT4 or metastatic (N/M1) tumors is fewer than 10% (17). Therefore, more effective systematic treatment is in urgent need for advanced UTUC patients.

Thus, we performed a retrospective study of Chinese UTUC patients to assess the prevalence of MMR deficiency and analyzed its clinicopathological implications. We also studied the efficacy of immunotherapy in advanced UTUC patients to explore more treatment regimens.

## Methods

### Patients and clinical information

Institutional Review Board approval was acquired at Fudan University Shanghai Cancer Center (FUSCC). All patients included in the study had signed written informed consent. A total of 175 patients with UTUCs were enrolled. Clinical data were retrospectively collected from the medical information system, including demographics, history and family history, pathological results, and radiographic imaging. We reviewed all patients in conformity with the standard of WHO classification, especially concerning pathological grading and tumor stage. In our study, we chose relapse-free survival (RFS) as the endpoint, which was defined from the time of initial surgery until recurrence and censored at death or last follow-up (18).

### Immunohistochemistry staining and evaluation

A total of 175 individual cases with formalin-fixed paraffin-embedded (FFPE) tumor blocks were collected. All hematoxylin and eosin-stained slides were reviewed by two professional pathologists. Tissue microarrays (TMAs) of 175 UTUC cases were constructed from the most typical regions of each case. MMR protein IHC was applied to detect the expression of the DNA MMR proteins MLH1 (#ab92312, Abcam, UK), PMS2 (#ab110638, Abcam, UK), MSH2 (#ab227941, Abcam, UK), and MSH6 (#ab92471, Abcam, UK). MMR proteins were considered lost when the tumor nuclei completely lacked staining and a positive internal control was present in the form of lymphocytes and/or endothelial cells.

### Statistical analysis

Variables were classified as continuous and categorical variables. Median (range) were used for continuous variables and frequencies were used for categorical factors. RFS was analyzed by using Kaplan–Meier analysis and different survival curves were compared *via* Log-rank test. We used Cox regression analysis to analyze the hazard ratio (HR) and 95% confidence interval (CI) for the association between different clinical factors and RFS. *p*-value < 0.05 was set to imply the level of statistical significance by using two-sided statistical tests. The statistical analyses were performed with SPSS Version 23.0 (IBM, Ehningen, Germany).

## Results

### Patient characteristics

In total, 175 patients with UTUC were analyzed in this study. Baseline characteristics are depicted in **Table 1**. The median age at diagnosis was 66 years old (range: 33 to 85 years), and 51.3% (*n* = 101) of the patients were male. In terms of laterality, 90 patients (51.4%) were left and 85 patients (48.6%) were right. A total of 57 patients (32.6%) were pT1 and 118 patients (67.7%) were pT2–pT4. Of 175 cases, high-grade UTUC (*n* = 163; 93.1%) was more prevalent than low-grade UTUC (*n* = 12; 6.9%). The tumor location was at the renal pelvis in 89 patients (50.9%), at the ureter in 72 patients (41.1%), and at both in 14 patients (8.0%).

**Table 1:** Baseline characteristics of the study cohort (*N* = 175).

Characteristics	Overall Cohort (*N*, %)	MMR protein loss (*N*, %)	No MMR protein loss (*N*, %)	*p*-value
Age at diagnosis, years				0.033
Median (range)	66 (33–85)	67 (52–80)	(33–85)	
Sex				0.611
Female	74 (37.6)	6 (31.6)	68 (43.6)	
Male	101 (51.3)	13 (68.4)	88 (56.4)	
Laterality				0.708
Left	90 (51.4)	9 (47.4)	81 (48.1)	
Right	85 (48.6)	10 (52.6)	75 (51.9)	
Smoking history				0.447
No	115 (65.7)	11 (57.9)	104 (66.7)	
Yes	60 (34.3)	8 (52.1)	52 (33.3)	
History of bladder cancer				**0.022**
No	156 (89.1)	14 (73.7)	142 (91.0)	
Yes	19 (10.9)	5 (26.3)	14 (9.0)	
History of non-bladder cancer				0.091
No	151 (86.3)	14 (73.7)	137 (87.8)	
Yes	24 (13.7)	5 (26.3)	19 (12.2)	
Family cancer history				0.405
No	134 (76.6)	16 (84.2)	118 (75.6)	
Yes	41 (23.4)	3 (15.8)	38 (24.4)	
T stage				0.332
T1	57 (32.6)	9 (47.4)	48 (30.8)	
T2	51 (29.1)	5 (26.3)	46 (29.5)	
T3	60 (34.3)	5 (26.3)	55 (35.3)	
T4	7 (4.0)	0 (0)	7 (4.5)	
N stage				0.425
N0	162 (92.6)	19 (100)	143 (91.7)	
N1	4 (2.3)	0 (0)	4 (2.6)	
N2	9 (5.1)	0 (0)	9 (5.8)	
Pathological stage				0.210
Low	12 (6.9)	0 (0)	12 (7.7)	
High	163 (93.1)	19 (100)	144 (92.3)	
Carcinoma *in situ*				0.163
No	155 (88.6)	16 (84.2)	139 (89.2)	
Yes	20 (11.4)	3 (15.8)	17 (10.8)	
Region				0.798
Renal pelvis	89 (50.9)	10 (52.6)	79 (50.6)	
Ureter	72 (41.1)	8 (42.1)	64 (41.0)	
Both	14 (8.0)	1 (5.3)	13 (8.3)	
MMR protein				/
Loss	19 (10.9)	19 (100)	0 (0)	
No loss	156 (89.1)	0 (0)	156 (100)	

Difference between patients with "MMR protein loss" and "No MMR protein loss" were compared, which was shown as P value. Bolded values indicate the P value<0.05, which was statistically significant.

### Expression profiles and clinicopathologic correlations of MMRs in UTUC

**Figure 1:**
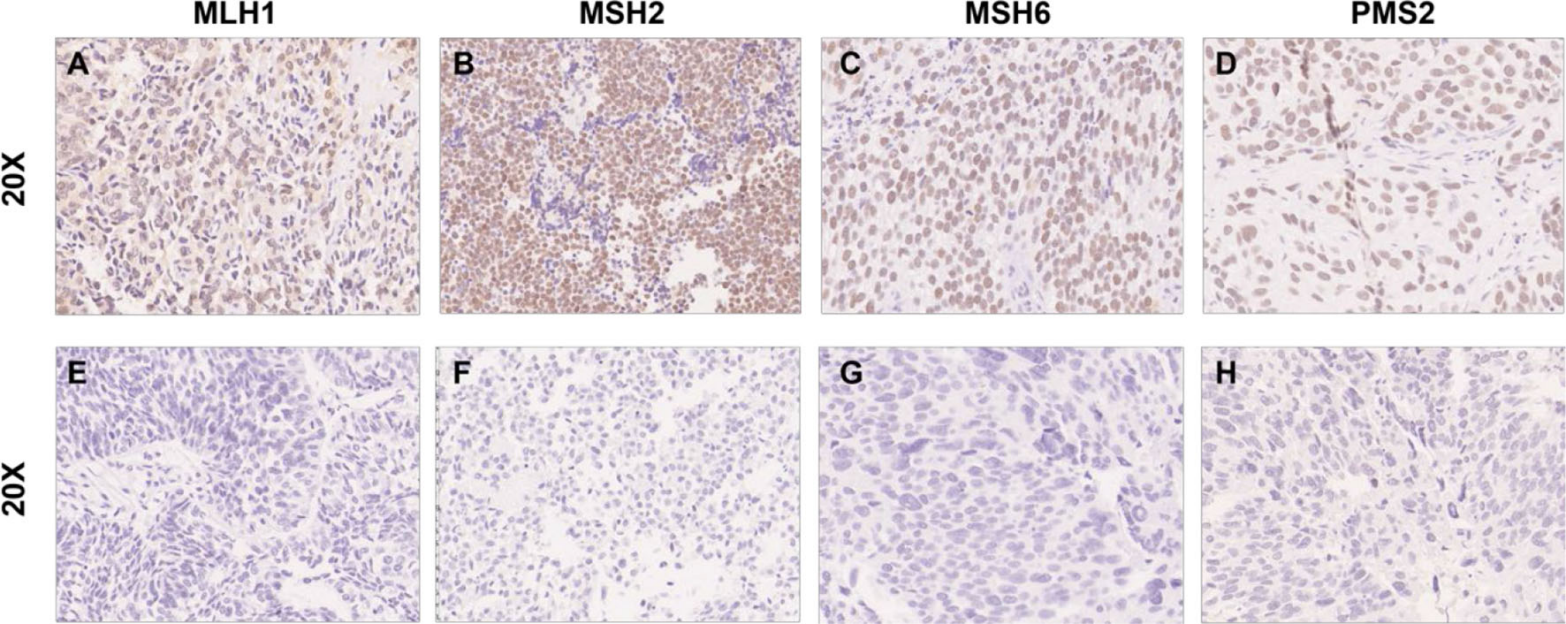
Representative image of negative/positive IHC results of MMR proteins.

We found loss of expression of at least one of the MMR proteins by IHC in 19 patients (10.9%). Six patients were found with the loss of both MSH2 and MSH6, two patients showed loss of MSH6 alone, six patients showed loss of MSH2 alone, three patients showed loss of both MLH1 and PMS2, and two patients showed PMS2 loss alone (**Figure 1**).

Clinical data of these 19 patients are demonstrated in **Table 2**. The median age was 66.7 years old (range: 52 to 80 years) and 13 cases (68.4%) were male. Ten cases were right and nine cases were left in terms of laterality. The tumor location was the renal pelvis in 10 cases, the ureter in 8 cases, and both in 1 case. All 19 patients showed high-grade tumors. Nine patients were pT1, five patients were pT2, and five patients were pT3. Three patients had a documented family cancer history, and five patients had UCB cancer history (**Table 2**).

**Table 2:** Data of patients with loss of MMR protein.

Case	Age	Sex	Laterality	Location	Grade	pT stage	Family cancer history	Loss pattern of MMR-IHC	UCB cancer history	Non-UCB cancer history
1	73	Male	Right	Renal pelvis	High	T3	No	MSH2, MSH6	No	No
2	66	Female	Left	Renal pelvis	High	T1	No	MSH6	Yes	No
3	80	Male	Right	Renal pelvis	High	T1	Brother, colon cancer	MSH2	No	No
4	56	Male	Left	Renal pelvis	High	T3	No	MSH2	No	No
5	68	Male	Right	Ureter	High	T1	No	MHL1, PMS2	Yes	Colon cancer
6	71	Male	Left	Both	High	T1	Father, colon cancer	MSH2, MSH6	No	Colon cancer
7	77	Male	Right	Ureter	High	T3	No	MSH2	No	No
8	69	Female	Left	Ureter	High	T1	No	MSH2, MSH6	No	No
9	52	Female	Left	Renal pelvis	High	T1	No	MSH2, MSH6	No	No
10	71	Female	Right	Ureter	High	T2	Father, lung cancer	MHL1, PMS2	No	Breast cancer
11	59	Male	Right	Renal pelvis	High	T1	No	MSH2	No	No
12	65	Male	Right	Renal pelvis	High	T2	No	MSH2, MSH6	No	No
13	61	Male	Right	Ureter	High	T1	No	MSH6	Yes	No
14	74	Male	Left	Renal pelvis	High	T3	No	MHL1, PMS2	Yes	No
15	66	Female	Left	Renal pelvis	High	T3	No	MSH2	No	No
16	71	Male	Left	Ureter	High	T2	No	MSH2	No	No
17	67	Male	Right	Renal pelvis	High	T2	No	MSH2, MSH6	No	Colon cancer
18	57	Male	Right	Ureter	High	T2	No	MHL1	Yes	No
19	65	Female	Left	Ureter	High	T1	No	MHL1	No	Thyroid cancer

### Impact of MMR expression on survival in UTUC

**Figure 2:**
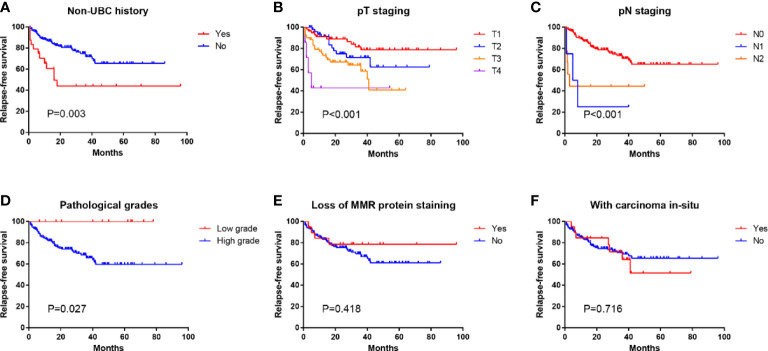
Kaplan–Meier analysis of relapse-free survival of patients with different **(A)** non-UBC history, **(B)** pT staging, **(C)** pN staging, **(D)** pathological grades, **(E)** MMR protein staining status, and **(F)** carcinoma *in situ* status.

Kaplan–Meier analysis showed that history of non-UBC (*p* = 0.003), pT staging (*p* < 0.001), pN staging (*p* < 0.001), and pathological grade (*p* = 0.027) were significant prognostic factors for RFS (**Figure 2**). However, it demonstrated that loss of MMR protein staining and carcinoma *in situ* were not significant prognostic factors for RFS. Univariate Cox regression analysis revealed that history of Non-UBC (*p* = 0.004), pT staging (*p* = 0.001), and pN staging (*p* = 0.001) were significant prognostic factors for RFS, which was further proven in multivariate Cox regression analysis (**Table 3**).

**Table 3:** Univariate and multivariate Cox regression analysis: relapse-free survival in upper urinary tract urothelial carcinoma.

Variables	Univariate analysis			Multivariate analysis		
	*p*	HR	95% CI	*p*	HR	95% CI
Age at diagnosis (≤65 vs. >65)	0.217	1.453	0.803–2.628			
Sex (Male vs. Female)	0.995	0.999	0.750–1.332			
Laterality (Right vs. Left)	0.917	1.015	0.765–1.347			
Smoking history (No vs. Yes)	0.977	0.991	0.549–1.791			
History of bladder cancer (No vs. Yes)	0.321	1.501	0.673–3.348			
History of non-bladder cancer (No vs. Yes)	**0.004**	2.612	1.357–5.028	**0.003**	2.783	1.408–5.500
Family cancer history (No vs. Yes)	0.889	0.954	0.496–1.836			
T stage	**0.001**			**0.015**	1.511	0.636–3.589
T2 vs. T1	0.246	1.648	0.709–3.832	0.349	2.555	1.192–5.479
T3 vs. T1	0.005	2.989	1.403–6.369	0.016	5.776	1.722–19.369
T4 vs. T1	<0.001	8.266	2.564–26.648	0.005		
N stage	**0.001**			**0.007**		
N1 vs. N0	0.006	5.287	1.626–17.194	0.021	4.276	1.248–14.656
N2 vs. N0	0.007	3.62	1.426–9.190	0.014	3.369	1.274–8.913
Pathological stage (Low vs. High)	0.153	23.658	0.308–1,819.740			
Carcinoma *in situ* (No vs. Yes)	0.717	1.16	0.520–2.589			
Region	0.485					
Ureter vs. Renal pelvis	0.385	1.296	0.722–2.326			
Ureter + Renal pelvis vs. Renal pelvis	0.556	0.696	0.208–2.326			
MMR protein (Loss vs. No loss)	0.422	1.521	0.546–4.234			

Bolded values indicate the P value<0.05, which was statistically significant, in the univariate analysis and multivariate analysis. HR, hazard ratio; CI, confidence interval; MMR protein, mismatch repair protein.

**Figure 3:**
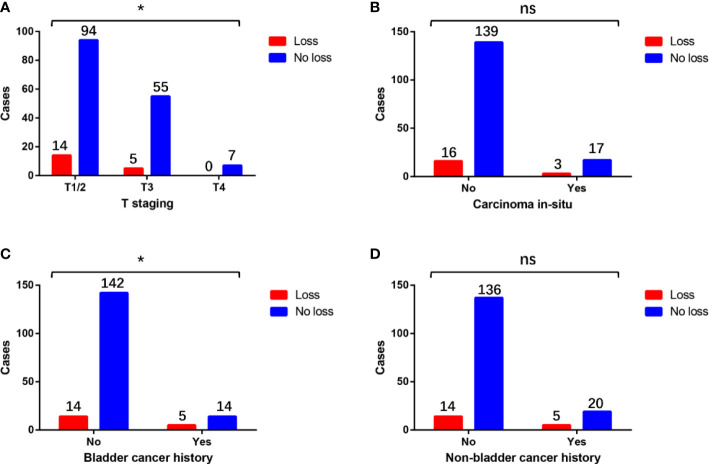
Differences in the distribution of the loss of MMR protein expression in patients with different **(A)** T staging, **(B)** carcinoma *in situ* status, **(C)** bladder cancer history, and **(D)** non-bladder cancer history. * means P<0.05, which is statistically significant. “ns” means not statistically significant.

There was no significant difference in terms of loss of MMR protein expression between cases with or without carcinoma *in situ* and non-bladder cancer history (**Figures 3B, D**). Compared with pT3 or pT4 patients, pT1–pT2 patients had a significantly higher proportion of the loss of MMR protein expression (**Figure 3A**). Compared with the patients without bladder cancer history, patients with bladder cancer history had a significantly higher proportion of the loss of MMR protein expression (**Figure 3C**).

### MMR expressions in patients developing advanced disease and receiving immunotherapy during follow-up

Distant or unresectable loco-regional recurrence was detected in 15 cases during a median follow‐up of 25 (range: 2–63) months after initial surgery. Information of these patients is provided in **Table 4**. Among them, two (13.3%) were loss of MMR expression. MMR IHC staining and radiographic images of two representative patients (one loss of MSH2 and one loss of MSH2) are shown in **Figure 3**. Ten patients received GC chemotherapy, and the other five received anti-PD-1 immunotherapy due to intolerance of or declination to chemotherapy.

**Table 4:** Characteristics of patients developing distant or unresectable locoregional recurrence during the follow‐up period (*N* = 15).

Characteristics	*N* (%)
Age at diagnosis, years	
Median (range)	69 (61–82)
Sex	
Female	4 (26.7)
Male	11 (73.3)
Unresectable recurrence site	
Lung metastases	11 (73.3)
Liver metastases	3 (20)
Unresectable local recurrence	1 (6.7)
Systematic therapy	
GC chemotherapy	10 (66.7)
Immunotherapy	5 (33.3)
MMR protein	
Loss	2 (13.3)
No loss	13 (86.7)
Duration from initial surgery to confirmation of the recurrence	
Median (range)	25 (2–63)
Alive/Dead	
Alive	10 (66.7)
Dead	5 (33.3)

We specifically checked detailed information of systematic treatment of two patients with loss of MMR protein. It demonstrated that case 1 (loss of MSH2 and MSH6) developed lung metastasis 3 months after the radical surgery. He then received three cycles of PD-1 inhibitors as immunotherapy. It demonstrated that the lesion in lung recessed a lot after the treatment. Case 7 (loss of MSH2) also developed lung metastasis 1 year after the surgery. This patient was also treated with PD-1 inhibitors. After six cycles of treatment, his lung metastasis also experienced a recession. Five patients (33.3%) died in the study period (**Table 4**).

## Discussion

UTUCs are gradually considered as a major extra-colon demonstration of LS. Patients suspected of LS have been assessed by using Amsterdam and Bethesda standards in the past (19–22). In fact, many patients at risk of LS cannot be identified by these two criteria. Encountering this problem, many institutions recommend universal screening for colorectal carcinoma (23) and endometrial carcinoma in these patients (24). Considering its high sensitivity, cost-effectiveness, and high availability, many medical institutions regard MMR-IHC as a first-line screening test for LS-associated cancer (13, 15). The prevalence of MMR IHC loss was 5% to 14% in UTUC cases according to recent studies (12, 13, 15, 25). Thus, Ju et al.^20^ suggested that universal LS screening test should be recommended in all UTUCs.

**Figure 4:**
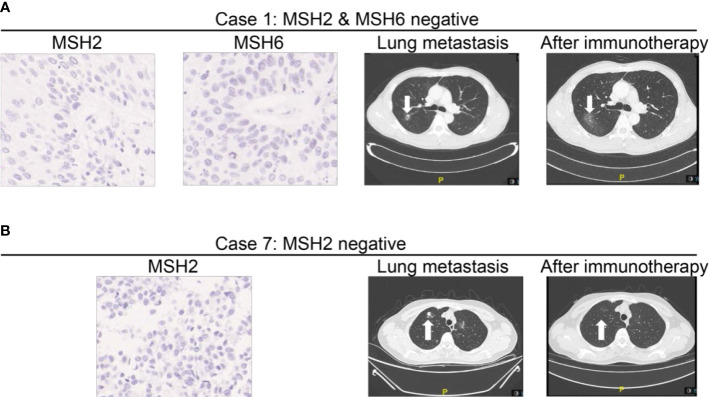
MMR expressions in patients developing advanced disease and receiving immunotherapy during follow-up. **(A)** Representative Case No. 1: A patient with loss of MSH2 and MSH6 staining developed lung metastasis after the surgery. After three cycles of PD-1 inhibitors as immunotherapy, the lesion in lung recessed a lot. **(B)** Representative Case No. 7: A patient with loss of MSH2 staining developed lung metastasis 1 year after the surgery. After 4 months of treatment, his lung metastasis also experienced a recession. Case 7 (loss of MSH2) also developed lung metastasis 1 year after the surgery. After six cycles of PD-1 inhibitors as immunotherapy, the lung metastasis also experienced a recession.

In our study, approximately 10.9% of cases were detected with loss of expression of the MMR proteins by IHC in UTUC. To date, this is the largest cohort study of unselected patients of UTUC for MMR protein testing in China. We found that the most common loss pattern was pairs of MSH2 and MSH6 (31.6%) as well as MSH2 alone (31.6%) in our study. According to recent studies, it is a well-known fact that the proportion of UTUC patients with MSH2/MSH6 loss (particularly MSH2 germline mutations) is more than patients with other types of MMR loss (15, 26). We found two representative cases among 19 patients, one case with both MSH2 loss and MSH6 loss and another case with MSH2 loss alone, and these two typical cases developed lung metastasis after radical surgery. After immunotherapy, lung lesion significantly recessed (**Figure 4**), which emphasized the predictive value of MMR loss for immunotherapy.

In our study, the average age of 19 patients with UTUC was 66.7 years, while the average age of the overall cohort cases was 66 years, which was very similar. The median age of LS patients with UTUC was lower; 52.6% of these locations are in the renal pelvis and 42.1% are in the ureter. All 19 of our LS-related UTUC patients had high-grade tumors in pathology, which was consistent with the high-level morphology of all UTUCs in LS patients in the previous literature (15).

Some limitations exist in our study. Although the sample size of 175 cases was already the largest cohort study in China, the single-center structure and retrospective nature still limited the generalization of this study. Therefore, further research is needed to verify our conclusions externally. In addition, we must also consider the disadvantages of TMA-based platforms. Even if the expression of MMR protein at the core of non-tumor tissue is misjudged positive for small tissue form, MMR loss may be underestimated due to sampling problems. Last but not least, we are unable to conduct genetic testing for all patients with MMR-IHC expression loss to achieve a final diagnosis. For this reason, our conclusions are still weak and need more in-depth analysis.

In conclusion, we studied the expression profile and clinicopathologic correlations of the MMR protein in the largest Chinese UTUC cohort, considering the rarity of UTUC and the relatively high incidence of LS-associated UTUC patients. In order to improve the cost-effectiveness and interest of patients and their families, we recommend MMR protein IHC (MLH1, PMS2, MSH2, and MSH6) to be included in the diagnostic guidelines for the general screening of LS-associated UTUC (27, 28).

## Data availability statement

The original contributions presented in the study are included in the article/Supplementary Material. Further inquiries can be directed to the corresponding authors.

Approval of the research protocol was acquired from the Institutional Reviewer Board of Fudan University Shanghai Cancer Center. Written informed consent was obtained from all participants for their participation in this study.

## Author contributions

ZS, JW and SJ analyzed the data and drafted the manuscript. WW, CG and YW helped interpreted the data. ZS, CY and YuZ prepared all figures, JW and SJ edited all tables. YS, YaZ and DY designed the study. All authors read and approved the final manuscript. 

## Funding

This work was supported by the National Natural Science Foundation of China (Grant Numbers 81972375, 81902568, 82172621, and 81702537), Shanghai Municipal Health Bureau (No. 2020CXJQ03), Clinical Research Plan of SHDC (SHDC2020CR4031), Shanghai Rising Star Program (Grant Number 16QA1401100), Shanghai “Rising Stars of Medical Talent” Youth Development Program, Shanghai Rising Star Program “Sailing” Project (Grant Number 22YF1408500), the General Program of Beijing Xisike Clinical Oncology Research Foundation (Grant Number Y-2019AZMS-0012), Shanghai Anti-Cancer Association Eyas Project (Grant Numbers SACA-CY19A01 and SACA-CY20A01), and Fudan University Shanghai Cancer Center Fund (No. YJQN202104).

## Acknowledgments

We sincerely thank all the study participants for their involvement in this research.

## Conflict of interest

The authors declare that the research was conducted in the absence of any commercial or financial relationships that could be construed as a potential conflict of interest.

## Publisher’s note

All claims expressed in this article are solely those of the authors and do not necessarily represent those of their affiliated organizations, or those of the publisher, the editors and the reviewers. Any product that may be evaluated in this article, or claim that may be made by its manufacturer, is not guaranteed or endorsed by the publisher.

## References

[1] Hong K Yao L Sheng X Ye D Guo Y. Neoadjuvant therapy of cyclin-dependent kinase 4/6 inhibitors combined with endocrine therapy in HR+/HER2- breast cancer: A systematic review and meta-analysis. Oncol Res Treat (2021) 44(10):557–67. doi: 10.1159/000518573 34515204

[2] Babjuk M Bohle A Burger M Capoun O Cohen D Comperat EM. EAU guidelines on non-muscle-invasive urothelial carcinoma of the bladder: Update 2016. Eur Urol (2017) 71(3):447–61. doi: 10.1016/j.eururo.2016.05.041 27324428

[3] Munoz JJ Ellison LM. Upper tract urothelial neoplasms: incidence and survival during the last 2 decades. J Urol (2000) 164(5):1523–5. doi: 10.1016/S0022-5347(05)67019-X 11025695

[4] Lynch HT Snyder CL Shaw TG Heinen CD Hitchins MP. Milestones of lynch syndrome: 1895-2015. Nat Rev Cancer (2015) 15(3):181–94. doi: 10.1038/nrc3878 25673086

[5] Lynch HT Lynch PM Lanspa SJ Snyder CL Lynch JF Boland CR. Review of the lynch syndrome: history, molecular genetics, screening, differential diagnosis, and medicolegal ramifications. Clin Genet (2009) 76(1):1–18. doi: 10.1111/j.1399-0004.2009.01230.x PMC2846640 19659756

[6] Tiwari AK Roy HK Lynch HT. Lynch syndrome in the 21st century: clinical perspectives. QJM (2016) 109(3):151–8. doi: 10.1093/qjmed/hcv137 26224055

[7] Evaluation of Genomic Applications in P Prevention Working G. Recommendations from the EGAPP working group: genetic testing strategies in newly diagnosed individuals with colorectal cancer aimed at reducing morbidity and mortality from lynch syndrome in relatives. Genet Med (2009) 11(1):35–41. doi: 10.1097/GIM.0b013e31818fa2ff 19125126 PMC2743612

[8] Giardiello FM Allen JI Axilbund JE Boland CR Burke CA Burt RW. Guidelines on genetic evaluation and management of lynch syndrome: a consensus statement by the US multi-society task force on colorectal cancer. Gastroenterology (2014) 147(2):502–26. doi: 10.1053/j.gastro.2014.04.001 25043945

[9] Watkins JC Yang EJ Muto MG Feltmate CM Berkowitz RS Horowitz NS. Universal screening for mismatch-repair deficiency in endometrial cancers to identify patients with lynch syndrome and lynch-like syndrome. Int J Gynecol Pathol (2017) 36(2):115–27. doi: 10.1097/PGP.0000000000000312 27556954

[10] Mochel MC Smith SC. Kidney tumors associated with hereditary cancer syndromes: an emerging opportunity and responsibility in surgical pathology. AJSP Rev Rep (2017) 22:313–28. doi: 10.1097/PCR.0000000000000220

[11] Ito T Kono K Eguchi H Okazaki Y Yamamoto G Tachikawa T. Prevalence of lynch syndrome among patients with upper urinary tract carcinoma in a Japanese hospital-based population. Jpn J Clin Oncol (2020) 50(1):80–8. doi: 10.1093/jjco/hyz140 31665498

[12] Metcalfe MJ Petros FG Rao P Mork ME Xiao L Broaddus RR. Universal point of care testing for lynch syndrome in patients with upper tract urothelial carcinoma. J Urol (2018) 199(1):60–5. doi: 10.1016/j.juro.2017.08.002 28797715

[13] Urakami S Inoshita N Oka S Miyama Y Nomura S Arai M. Clinicopathological characteristics of patients with upper urinary tract urothelial cancer with loss of immunohistochemical expression of the DNA mismatch repair proteins in universal screening. Int J Urol (2018) 25(2):151–6. doi: 10.1111/iju.13481 29164703

[14] Marshall FF. Urothelial carcinoma of the renal pelvis: a clinicopathologic study of 130 cases. J Urol (2005) 174(4 Pt 1):1249. doi: 10.1097/01.ju.0000175929.85043.51 16145381

[15] Harper HL McKenney JK Heald B Stephenson A Campbell SC Plesec T. Upper tract urothelial carcinomas: frequency of association with mismatch repair protein loss and lynch syndrome. Modern Pathol an Off J U States Can Acad Pathol Inc (2017) 30(1):146–56. doi: 10.1038/modpathol.2016.171 27713421

[16] Yates DR Catto JW. Distinct patterns and behaviour of urothelial carcinoma with respect to anatomical location: how molecular biomarkers can augment clinico-pathological predictors in upper urinary tract tumours. World J Urol (2013) 31(1):21–9. doi: 10.1007/s00345-012-0946-6 22986906

[17] Lughezzani G Burger M Margulis V Matin SF Novara G Roupret M. Prognostic factors in upper urinary tract urothelial carcinomas: a comprehensive review of the current literature. Eur Urol (2012) 62(1):100–14. doi: 10.1016/j.eururo.2012.02.030 22381168

[18] Suciu S Eggermont AMM Lorigan P Kirkwood JM Markovic SN Garbe C. Relapse-free survival as a surrogate for overall survival in the evaluation of stage II-III melanoma adjuvant therapy. J Natl Cancer Inst (2018) 110(1). doi: 10.1093/jnci/djx133 28922786

[19] Vasen HF Mecklin JP Khan PM Lynch HT. The international collaborative group on hereditary non-polyposis colorectal cancer (ICG-HNPCC). Dis Colon Retum (1991) 34(5):424–5. doi: 10.1007/BF02053699 2022152

[20] Vasen HF Watson P Mecklin JP Lynch HT. New clinical criteria for hereditary nonpolyposis colorectal cancer (HNPCC, lynch syndrome) proposed by the international collaborative group on HNPCC. Gastroenterology (1999) 116(6):1453–6. doi: 10.1016/S0016-5085(99)70510-X 10348829

[21] Boland CR Thibodeau SN Hamilton SR Sidransky D Eshleman JR Burt RW. A national cancer institute workshop on microsatellite instability for cancer detection and familial predisposition: development of international criteria for the determination of microsatellite instability in colorectal cancer. Cancer Res (1998) 58(22):5248–57. 9823339

[22] Umar A Boland CR Terdiman JP Syngal S de la Chapelle A Ruschoff J. Revised Bethesda guidelines for hereditary nonpolyposis colorectal cancer (Lynch syndrome) and microsatellite instability. J Natl Cancer Inst (2004) 96(4):261–8. doi: 10.1093/jnci/djh034 PMC2933058 14970275

[23] Musulen E Sanz C Munoz-Marmol AM Ariza A. Mismatch repair protein immunohistochemistry: a useful population screening strategy for lynch syndrome. Hum Pathol (2014) 45(7):1388–96. doi: 10.1016/j.humpath.2014.02.012 24768606

[24] Mills AM Sloan EA Thomas M Modesitt SC Stoler MH Atkins KA. Clinicopathologic comparison of lynch syndrome-associated and "Lynch-like" endometrial carcinomas identified on universal screening using mismatch repair protein immunohistochemistry. Am J Surg Pathol (2016) 40(2):155–65. doi: 10.1097/PAS.0000000000000544 26523542

[25] Olagui GS Pignot G Rouquette A Vieillefond A Amsellem-Ouazana D de Longchamps NB. [Should we systematically screen for lynch syndrome in patients with upper urinary tract carcinoma?]. Bull Cancer (2014) 101(2):144–50. doi: 10.1684/bdc.2014.1896 24556207

[26] Acher P Kiela G Thomas K O'Brien T. Towards a rational strategy for the surveillance of patients with lynch syndrome (hereditary non-polyposis colon cancer) for upper tract transitional cell carcinoma. BJU Int (2010) 106(3):300–2. doi: 10.1111/j.1464-410X.2010.09443.x 20553255

[27] Daniels MS. Genetic testing by cancer site: uterus. Cancer J (2012) 18(4):338–42. doi: 10.1097/PPO.0b013e3182610cc2 22846735

[28] Ju JY Mills AM Mahadevan MS Fan J Culp SH Thomas MH. Universal lynch syndrome screening should be performed in all upper tract urothelial carcinomas. Am J Surg Pathol (2018) 42(11):1549–55. doi: 10.1097/PAS.0000000000001141 30148743

